# Isolation of PCSK9-specific nanobodies from synthetic libraries using a combined protein selection strategy

**DOI:** 10.1038/s41598-025-88032-1

**Published:** 2025-01-28

**Authors:** Apisitt Thaiprayoon, Yodpong Chantarasorn, Worrapoj Oonanant, Anongnard Kasorn, Phoomintara Longsompurana, Satita Tapaneeyakorn, Pinpunya Riangrungroj, Fabien Loison, Andrew C. Kruse, Matthew P. DeLisa, Dujduan Waraho-Zhmayev

**Affiliations:** 1 Biological Engineering Program, Faculty of Engineering, King Mongkut’s University of Technology Thonburi, Bangkok, 10140 Thailand; 2 Division of Ophthalmology, Faculty of Medicine, Vajira Hospital, Navamindradhiraj University, Bangkok, Thailand; 3 Department of Basic Medical Science, Faculty of Medicine Vajira Hospital, Navamindradhiraj University, Dusit, Bangkok, Thailand; 4 National Nanotechnology Center (NANOTEC), National Science and Technology Development Agency (NSTDA), Thailand Science Park, Khlong Luang, Pathumthani 12120 Thailand; 5 National Center for Genetic Engineering and Biotechnology (BIOTEC), National Science and TechnologyDevelopment Agency (NSTDA), 111 Thailand Science Park, Phahonyothin Road, Klong Nueng, Klong Luang, Pathumthani 12120 Thailand; 6 Department of Microbiology, Faculty of Science, Mahidol University, Bangkok, Thailand; 7 Department of Biological Chemistry and Molecular Pharmacology, Harvard Medical School, Boston, MA 02115 USA; 8 Robert F. Smith School of Chemical and Biomolecular Engineering, Cornell University, Ithaca, NY 14853 USA; 9 Cornell Institute of Biotechnology, Cornell University, Ithaca, NY 14853 USA

**Keywords:** Camelid antibody, Genetic selection, High-throughput screening, Hypercholesterolemia, Synthetic antibody library, VHH single-domain antibody, Biochemistry, Drug discovery, Molecular biology, Genetic engineering, High-throughput screening, Microbiology techniques, Molecular engineering

## Abstract

Nanobodies (Nbs) hold great potential to replace conventional antibodies in various biomedical applications. However, conventional methods for their discovery can be time-consuming and expensive. We have developed a reliable protein selection strategy that combines magnetic activated cell sorting (MACS)-based screening of yeast surface display (YSD) libraries and functional ligand-binding identification by Tat-based recognition of associating proteins (FLI-TRAP) to isolate antigen-specific Nbs from synthetic libraries. This combined process enabled isolation of three unique Nb clones (NbT15, NbT21, and NbT22) that all bound specifically to a target antigen, namely proprotein convertase subtilisin/kexin type 9 (PCSK9) as well as a gain-of-function PCSK9 mutant (D374Y). All three clones bound to PCSK9 and blocked the interaction between the low-density lipoprotein receptor (LDLR) and either wild-type PCSK9 or the D374Y mutant. Overall, our combined protein selection method enables rapid and straightforward identification of potent antigen-specific Nbs in a manner that can be executed in a basic laboratory setting without the need for specialized equipment. We anticipate that our strategy will be a valuable addition to the protein engineering toolkit, allowing development of Nbs or virtually any other synthetic binding protein for a wide range of applications.

## Introduction

The biologics market is among the fastest-growing segments within the pharmaceutical industry with over 100 antibody-based therapeutics now approved by the U.S. Food and Drug Administration (FDA) and/or the European Medicines Agency (EMA)^1^. While the most common format is the full-length monoclonal antibody (mAb), other formats have also gained approval including fragments derived from mAbs such as fragment antigen-binding region (Fab) and single-chain variable fragment (scFv) antibodies as well as single-domain antibodies (sdAbs) from camelid species^2^. These sdAbs, also known as VHHs or nanobodies (Nbs) are the smallest single antigen-binding domains derived from dromedary species such as camels, llamas, or alpacas. Since the FDA approved the first therapeutic Nb (caplacizumab) in February 2019, they have become highly promising in preclinical and clinical settings due to their favorable pharmacological properties and versatility in therapeutic design. Nbs possess a longer complementarity determining region 3 (CDR3) that is important for antigen recognition and can confer binding to hidden epitopes that are inaccessible to mAbs^3^. Moreover, the small size of Nbs enables tolerance to harsh environments and permits expression as a soluble protein using microbial expression systems^4^. Altogether, these properties of Nbs offer significant advantages in terms of engineerability and manufacturing relative to their mAb counterparts.

To discover novel antigen-specific antibodies, a variety of technologies have been developed over the years^5^. One of the most versatile approaches for antibody discovery is surface display technology, in which the antibody is physically presented on the surface of phage, bacteria, yeast, or mammalian cells and is thus made available for antigen binding in vitro. By surface displaying a library of antibodies (i.e., repertoire of naïve, synthetic, or immune-focused antibody clones), antigen-specific binders can often be isolated following several rounds of biopanning against the antigen of interest. However, one drawback of these display methods is the occurrence of misfolding and expression-level biases of the antibody clones, which can lead to unwanted false-positive protein selections^6^. Moreover, the screening of cell surface display libraries typically involves the use of fluorescence-activated cell sorting (FACS), which is expensive and may not be accessible to many molecular biology laboratories^7^.

One method that overcomes some of these issues is a bacterial genetic selection method called FLI-TRAP (functional ligand-binding identification by twin-arginine translocation (Tat)-based recognition of associating proteins)^8^. FLI-TRAP exploits the bacterial Tat pathway, a protein translocation pathway that exports already folded proteins from the cytoplasm to the periplasm^9^. In addition to exporting folded monomeric proteins, the Tat system can also tolerate export of heterodimeric and -trimeric complexes via a unique “hitchhiker” mechanism in which two or three proteins assemble in the cytoplasm and are subsequently exported to the periplasm by virtue of the Tat signal peptide on one of the proteins^8,10^. To convert this mechanism into a genetic selection for discovering and engineering binding proteins, we genetically fused the Tat-dependent signal peptide of *Escherichia coli* (*E. coli*) trimethylamine-N-oxide reductase 1 (ssTorA) to a binding protein of interest (X). In parallel, we genetically fused the binding partner or target antigen (Y) to the β-lactamase (Bla) enzyme but without any signal peptide. When the ssTorA-X and Y-Bla constructs were co-expressed in the cytoplasm of *E. coli*cells, the interaction between X and Y triggered the formation of a ssTorA-X::Y-Bla heterodimeric complex, which was exported to the periplasm by the endogenous Tat transport system^8^. The co-translocation of the signal peptide-less Y-Bla construct into the periplasm rendered the cells resistant to β-lactam antibiotics such as ampicillin (Amp) and carbenicillin (Carb). Based on the observation that the level of antibiotic resistance of the cells correlates positively with the strength of the interaction between the X and Y proteins, this technique has been used to evolve antibody fragments with improved affinity^8^. A major advantage of this method is its simplicity, requiring inexpensive reagents and supplies that are widely available in most molecular biology laboratories.

In this study, we attempted to develop a straightforward strategy for isolating Nbs against targets of interest. For the target antigen, we chose the catalytic (CAT) domain of human proprotein convertase subtilisin/kexin type 9 (PCSK9). PCSK9 is a zymogen comprised of three domains: a pro-domain, a CAT domain, and a C-terminal histidine-rich domain. PCSK9 cleaves its pro-domain in the endoplasmic reticulum to form mature PCSK9, which is then secreted into the bloodstream where it binds the low-density lipoprotein receptor (LDLR)^11^. This binding prevents the LDLR from forming a closed conformation, inhibiting its recycling process and leading to its lysozyme-mediated degradation. As a result of this activity, PCSK9 is a major cause of hypercholesterolemia (i.e., high cholesterol levels in the blood)^12,13^. To isolate Nbs against PCSK9, we executed a straightforward two-step procedure. In the first step, yeast cells expressing a fully synthetic Nb library on their surface^14^ were pre-screened for antigen binding by a single round of magnetic activated cell sorting (MACS). Then, in the second step, an antigen-focused sublibrary was subjected to a single round of genetic selection using FLI-TRAP to isolate individual clones with high specificity for PCSK9. Using this approach, we isolated three clones that bound specifically to PCSK9. Importantly, these clones also bound tightly to PCSK9^D374Y^, a gain-of-function mutant that is known to increase total cholesterol by around 33% due to its stronger affinity for LDLR and in turn increases the development of familial hypercholesterolemia^15,16^. These newly identified Nbs hold promise for their ability to target PCSK9 and could prove useful as treatments for hypercholesterolemia in the future.

**Fig. 1:**
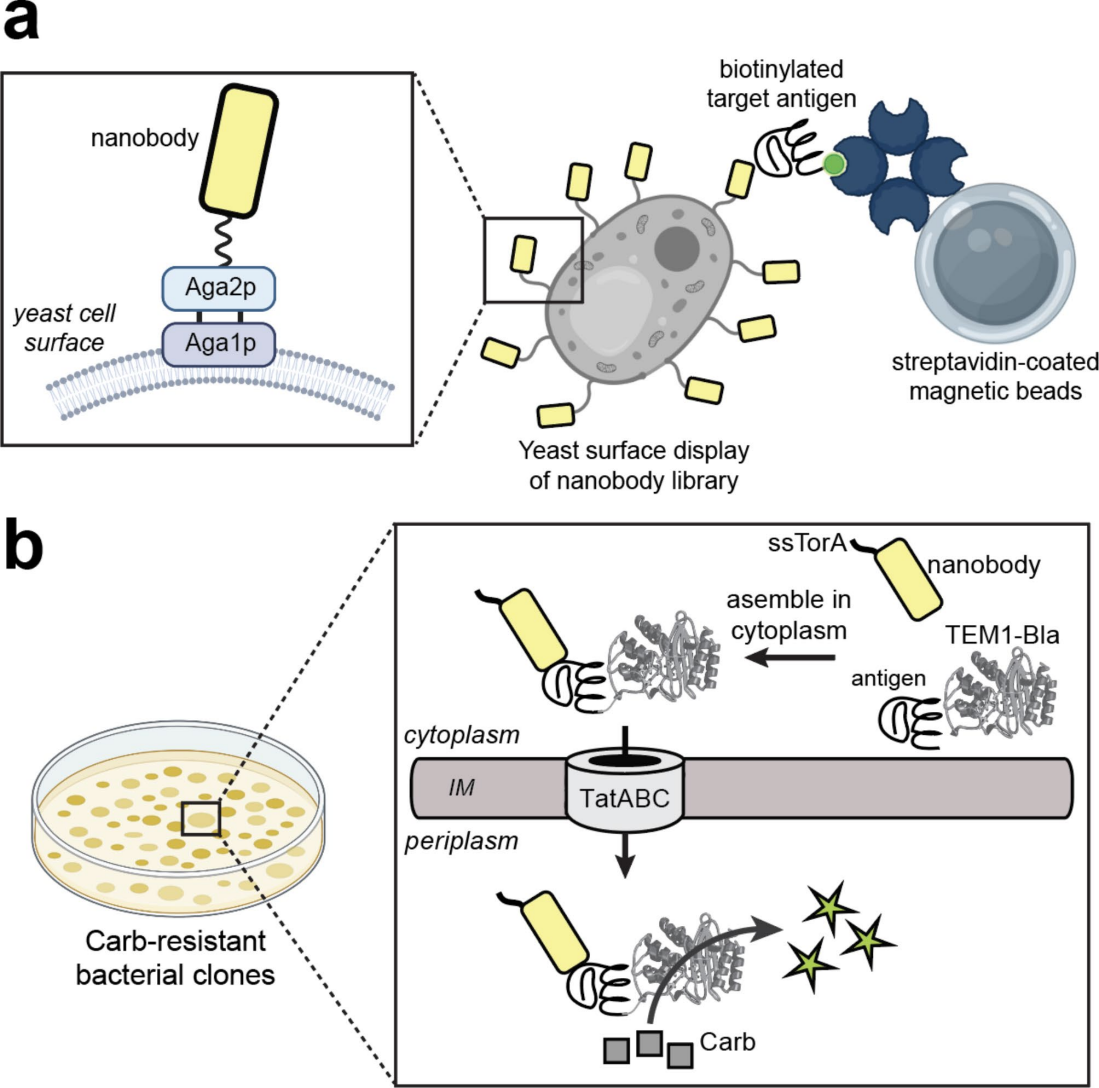
Schematic of the combined strategy for isolating PCSK9-specific Nbs. (**a**) Display of the synthetic nanobody library on yeast cells and screening for PCSK9-specific Nbs via MACS-based yeast surface display. (**b**) Isolation of individual Carb-resistant bacterial clones using FLI-TRAP selection as described in the text.

## Results and discussion

### A combined strategy for isolating PCSK9 binders from a synthetic Nb library

For the first step in our combined strategy, we leveraged a fully synthetic library that was previously formatted for yeast surface display (YSD)^14^ (Fig. 1). This fully synthetic library was developed to increase the variation of the CDR regions via bioengineering such that a more diverse library could be constructed without the concern of producing self-binding hits. Advantages of this large synthetic library relative to an immune-focused library are that it enables antibody selection against virtually unlimited targets using the same library and it sidesteps concerns about immunological tolerance. Whereas conventional YSD involves two steps, MACS and fluorescence activated cell sorting (FACS), in this study we performed only a single round of MACS to reduce the size of the initial Nb library and focus the library members to the PCSK9 protein. Specifically, a YSD library containing ~ 5 × 10^8^ unique Nb clones was subjected to biopanning with magnetic beads coated with full-length PCSK9.

For the second step in our combined strategy, a sublibrary of positively selected clones from the YSD screening step above were incorporated into the FLI-TRAP system. Specifically, genes encoding the YSD-selected clones were amplified by PCR and the PCR products were cloned into the pDD18-Cm ssTorA-Nb-FLAG plasmid. The resulting plasmid library encoded the YSD-selected Nbs fused with a Tat-dependent signal peptide at their N-termini and a FLAG epitope tag at their C-termini. In parallel, we constructed plasmid pDD322-CAT-Bla, which encoded a fusion protein composed of the PCSK9 CAT domain and the Bla reporter protein. The rationale for the selection strategy is that productive interaction between an ssTorA-Nb-FLAG clone and CAT-Bla forms a heterodimeric ssTorA-Nb::CAT-Bla complex, which is subsequently translocated to the periplasmic space via the Tat pathway and renders the bacterial host cell resistant to β-lactam antibiotics (Fig. 1).

**Fig. 2:**
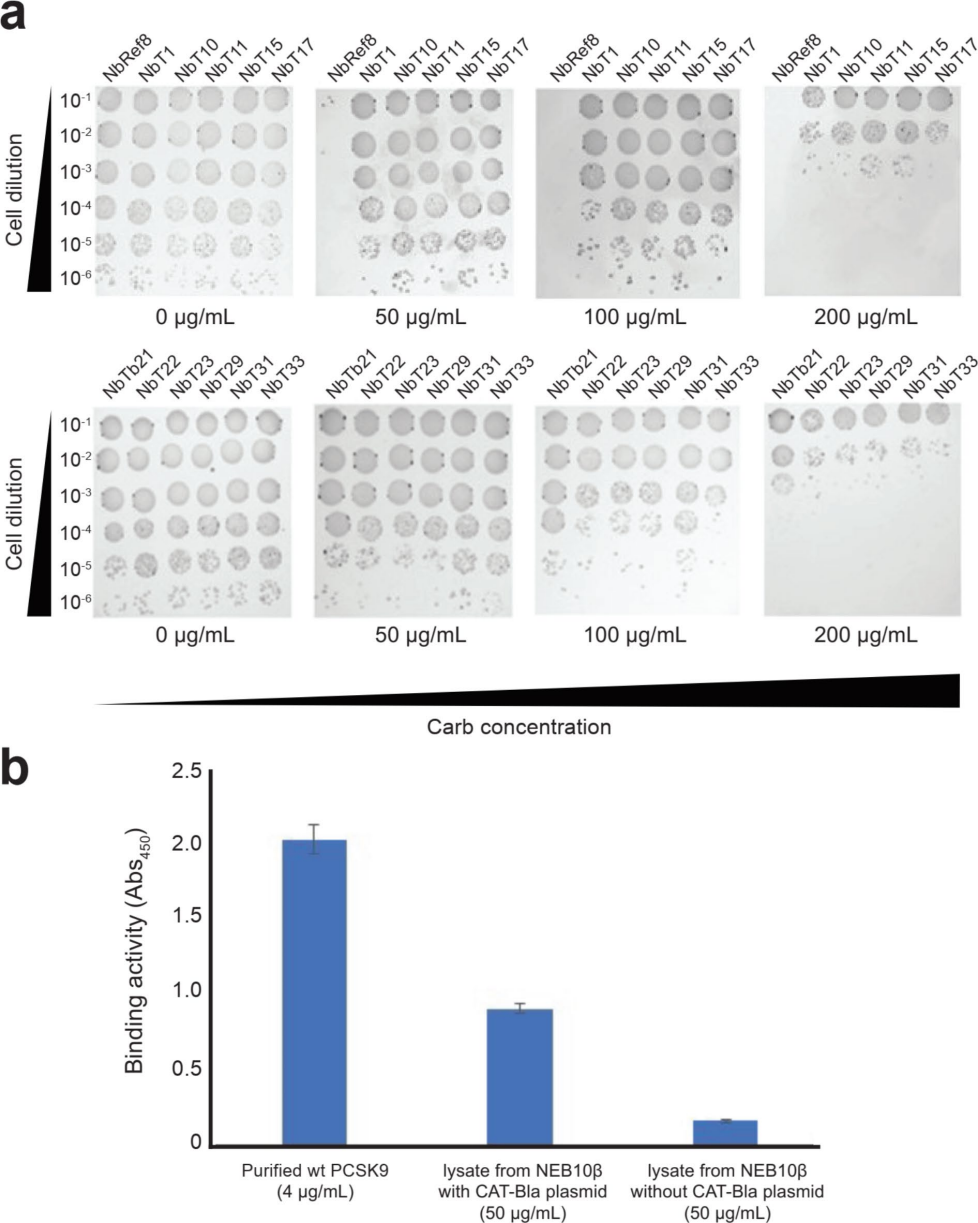
Selection of PCSK9 CAT domain-specific Nbs via FLI-TRAP. (a) Serial dilutions of randomly selected *E. coli* NEB10β cells expressing PCSK9-specific Nbs were spotted on plates containing 0–200 µg/mL of Carb to select PCSK9-specific Nbs. (**b**) ELISA analysis of cell lysates from NEB10β cells with and without CAT-Bla. Plates were coated with HEK293-derived LDLR as immobilized antigen and purified wild-type (wt) PCSK9 served as positive control. Data are the average of biological replicates (*n =* 3) ± standard deviation.

To select PCSK9 CAT domain-specific Nbs, *E. coli* NEB10β cells were transformed with both plasmids described above and selected on LB-agar plates containing 300 µg/mL Carb. We chose this concentration because resistance of *E. coli*cells to a high concentration of Carb represents a strong interaction between the putative protein partners^17^. A total of 30 Carb-resistant colonies that maintained both plasmids were selected under these conditions. DNA sequencing analysis of the 30 hits revealed 11 unique, full-length Nb sequences (the remaining 19 were either truncation sequences that contained a stop codon within the Nb or duplicate sequences). To confirm the ability of each full-length Nb to bind the target antigen and render cells resistant to Carb, we performed spot plating analysis. As expected, all 11 FLI-TRAP-selected clones conferred strong resistance on 50 and 100 µg/mL Carb, with measurable but reduced resistance at 200 µg/mL Carb (Fig. 2a). We also included Ref8, an Nb randomly chosen from a non-selected library that was used as a negative control based on its lack of binding to the PCSK9 CAT domain. As expected, the ssTorA-Ref8-FLAG construct was incapable of conferring Carb resistance to cells under the conditions tested, with cell growth inhibited at the lowest concentration of Carb (50 µg/mL) used here.

To confirm that the PCSK9 CAT domain in our CAT-Bla chimera was produced as a soluble, correctly folded protein, we performed enzyme-linked immunosorbent assay (ELISA) with commercial LDLR purified from human HEK293 cells as immobilized antigen. Notably, whole cell lysate of *E. coli* NEB10β cells expressing CAT-Bla showed a strong interaction, while the lysate from plasmid-free *E. coli* NEB10β cells showed only a weak signal (Fig. 2b).

### FLI-TRAP-selected Nbs specifically bind to wt and mutant PCSK9

**Fig. 3:**
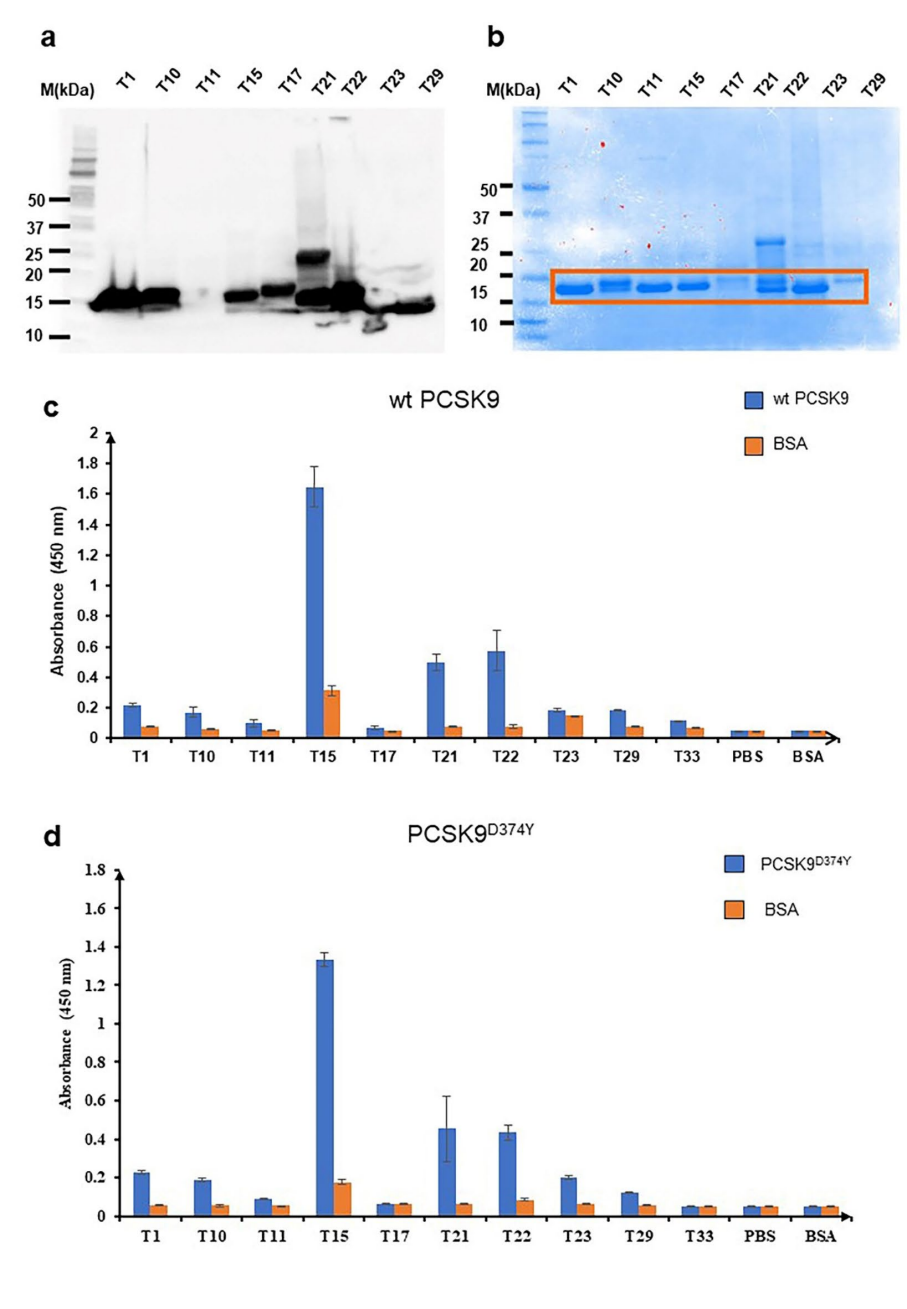
Expression and binding activity of FLI-TRAP selected Nb clones. (**a**) Western blot analysis of lysates derived from *E. coli* BL21(DE3) cells expressing each of the Nbs from plasmid pET28. (**b**) SDS-PAGE analysis of purification of Nbs following purification by Ni-NTA column chromatography. ELISA analysis of the purified Nbs using (**c**) wt PCSK9 and (**d**) gain-of-function PCSK9^D374Y^ mutant as immobilized antigens (blue bars). In both cases, 20 µg/mL of purified Nbs were added to the plate and immobilized bovine serum albumin (BSA) served as negative control (orange bars). Data are the average of biological replicates (*n =* 3) ± standard deviation.

 Since the Nbs were selected in the cytoplasm of *E. coli* cells for binding to the PCSK9 CAT domain, it was essential to confirm that they also bind to native, full-length PCSK9. To this end, we successfully expressed and purified nine Nbs, which ranged in size from 15 to 17 kDa, as observed from Western blot and SDS-PAGE analysis as well as primary sequence prediction using the ExPASy Server, due to differences in their CDR3 lengths (12–20 amino acid residues) (Fig. 3a-b). The binding activities of these solubly expressed Nbs were measured by ELISA using full-length PCSK9 produced in HEK293 cells as immobilized antigen. ELISA-based screening of the nine Nb clones revealed that six Nbs (NbT1, NbT10, NbT15, NbT21, NbT22, and NbT29) exhibited specific binding to wt PCSK9 with signals that were more than three-fold above the binding to a non-specific antigen, bovine serum albumin (BSA) (Fig. 3c). NbT15 showed the greatest binding to PCSK9 while NbT22 and NbT21 each showed weaker binding that was still significantly above background binding to the non-specific control. Hence, these three clones were chosen for further characterization. Importantly, the confirmation of seven PCSK9-CAT-specific Nbs (clone Nbt22 was isolated twice) out of 30 isolated clones indicates a 23% positive hit rate for our combined selection strategy.

**Fig. 4:**
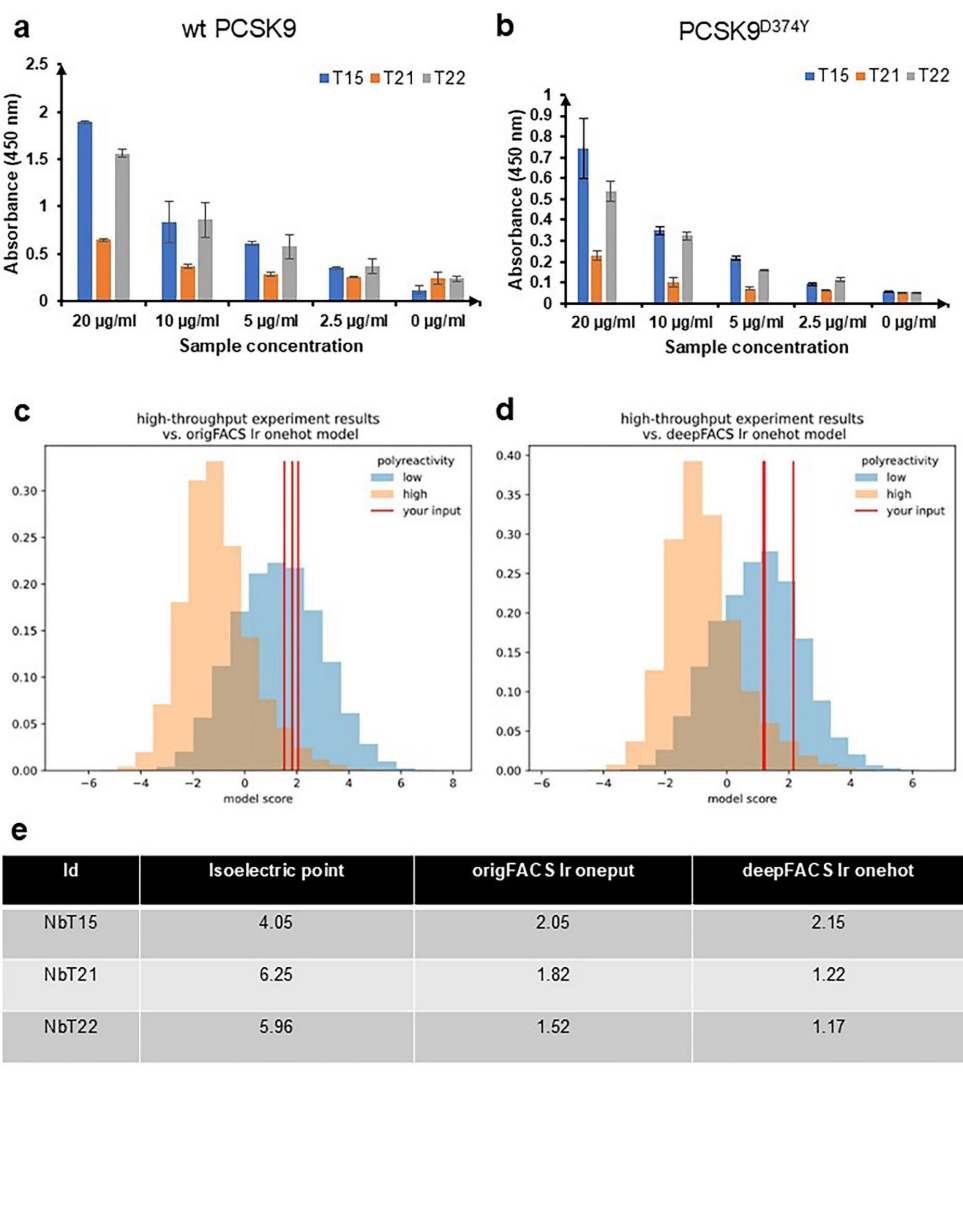
Detailed binding characterization of NbT15, NbT21, and NbT22 clones. (**a**, **b**) ELISA analysis of varying concentrations of NbT15 (blue), NbT21 (orange), and NbT22 (gray) with either wt PCSK9 or PCSK9^D374Y^ as immobilized antigen. Data are the average of biological replicates (*n =* 3) ± standard deviation. (**c**, **d**) The oriFACS lr one-hot model and deepFACS lr one-hot model of three Nbs from one-hot and three-mer logistic regression models. (**e**) Summary of three Nbs polyreactivity predictions.

We next investigated binding of the Nbs to PCSK9^D374Y^, a gain-of-function mutant that has 6–30-fold greater affinity for LDLR^16^. As a result of its higher binding affinity, PCSK9^D374Y^ inhibits LDLR recycling more strongly and thus promotes 33% greater lysozyme-mediated degradation. Importantly, NbT15, NbT21, and NbT22 all bound to PCSK9^D374Y^ to an extent that rivaled their individual binding to wt PCSK9 (Fig. 3d). Moreover, all three Nbs showed dose-dependent binding against wt and PCSK9^D374Y^ (Fig. 4a, b).

In addition, polyreactivity scores were computationally predicted for all three selected Nbs using the one-hot and 3-mer logistic regression models. The blue area in the oriFACs and deepFACs lr one-hot models correlates with the low polyreactivity model, while the orange area correlates with high polyreactivity. The NbT15, NbT21, and NbT22 Nbs had oriFACs lr one-hot model predictions of 2.05, 1.82, and 1.53, respectively, and deepFACs lr one-hot predictions of 2.15, 1.22, and 1.17, respectively (Fig. 4c-e**)**. The oriFACs and deepFACs LR one-hot model scores indicated that all Nbs have low polyreactivity (i.e., they were > 1). All three selected Nbs may have shown low polyreactivity because they were isolated in an in vivo environment containing native cytoplasmic *E. coli* proteins. We hypothesized that any Nbs with high polyreactivity might bind to non-target proteins, resulting in lower cell resistance to Carb due to lower Tat transport of the ssTorA-Nb::CAT-Bla complex into the periplasm.

### Isolated Nbs neutralize the interaction between PCSK9 and LDLR

**Fig. 5:**
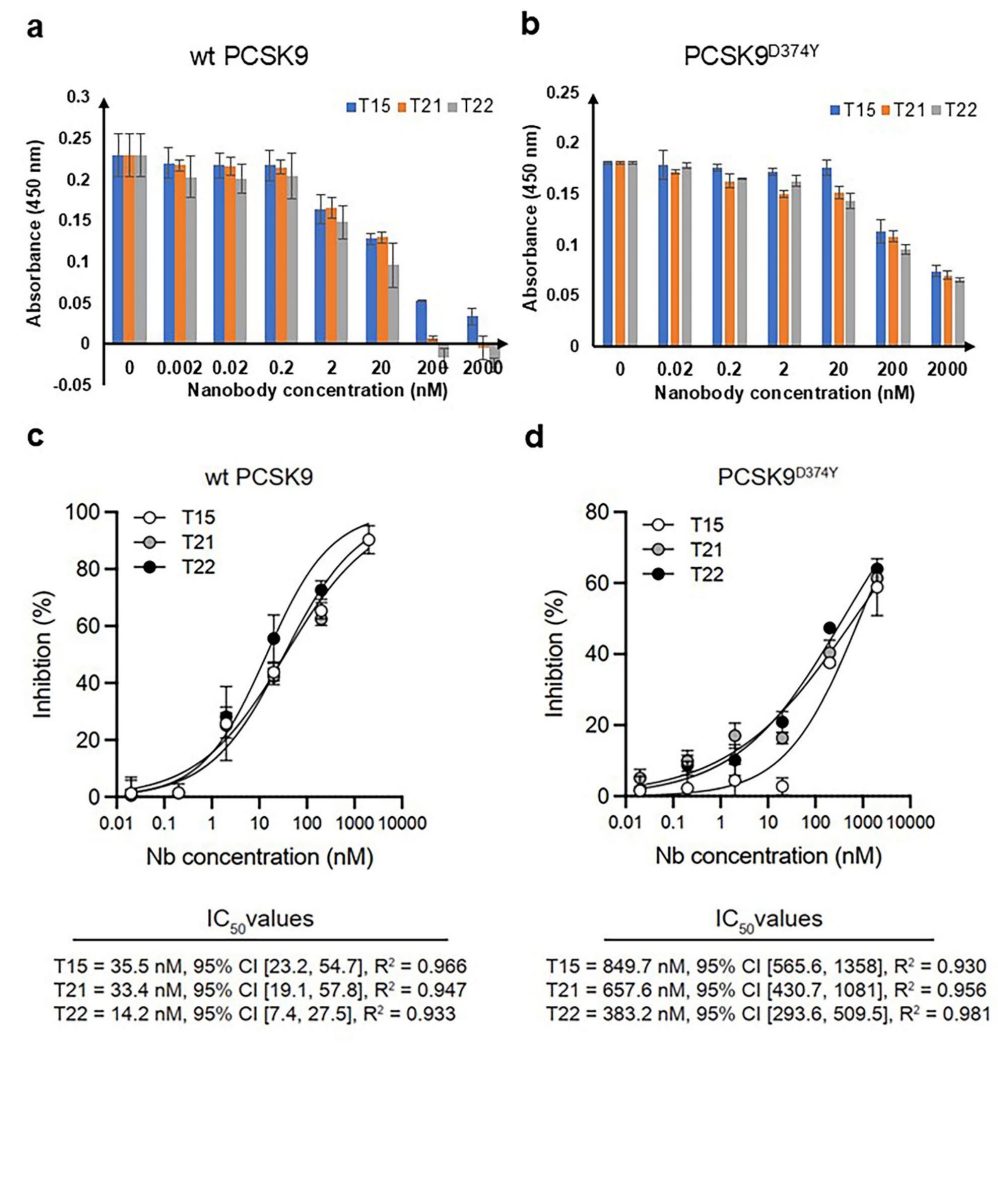
Inhibition of interaction between LDLR and wt PCSK9 or PCSK9^D374Y^by selected Nbs. (**a**, **b**) Neutralization activity of NbT15, NbT21, and NbT22 against the interaction between LDLR and wt PCSK9. Data are the average of biological replicates (*n =* 3) ± standard deviation. (**c**) Summary of three Nbs IC_50_ as calculated using GraphPad Prism software.

 We next determined whether any of the isolated Nbs could inhibit the binding of PCSK9 to LDLR using a modified ELISA strategy. Importantly, all three selected Nbs inhibited the interaction between the LDLR and both wt PCSK9 and PCSK9^D374Y^ in a dose-dependent manner (Fig. 5a, b). The data clearly shows the following: (1) all Nbs inhibit wt PCSK9 more potently than the PCSK9^D374Y^ and (2) NbT15 and NbT21 are nearly identical in their inhibition while NbT22 does appear to be a more potent inhibitor (95% CI) (Fig. 5c-d).

**Fig. 6:**
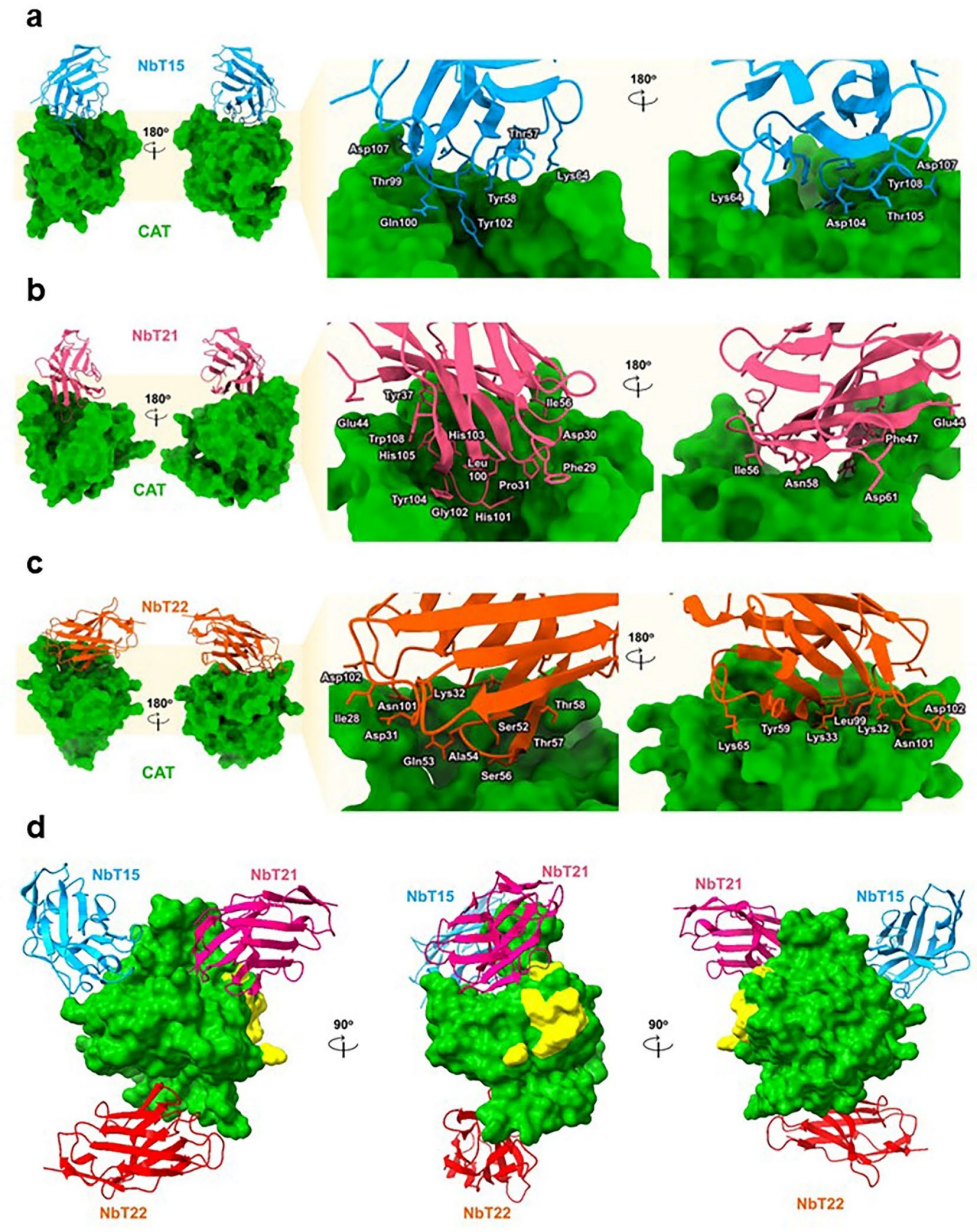
Predicted amino acid interactions between isolated Nbs and wt PCSK9. The interactions of (**a**) NbT15, (**b**) NbT21, and (**c**) NbT22 with wt PCSK9. (**d**) The NbT15, NbT21, and NbT22 binding sites on the PCSK9 CAT domain. Yellow area highlights the amino acid residues of the epidermal growth factor-like repeat A binding domain.

**Fig. 7:**
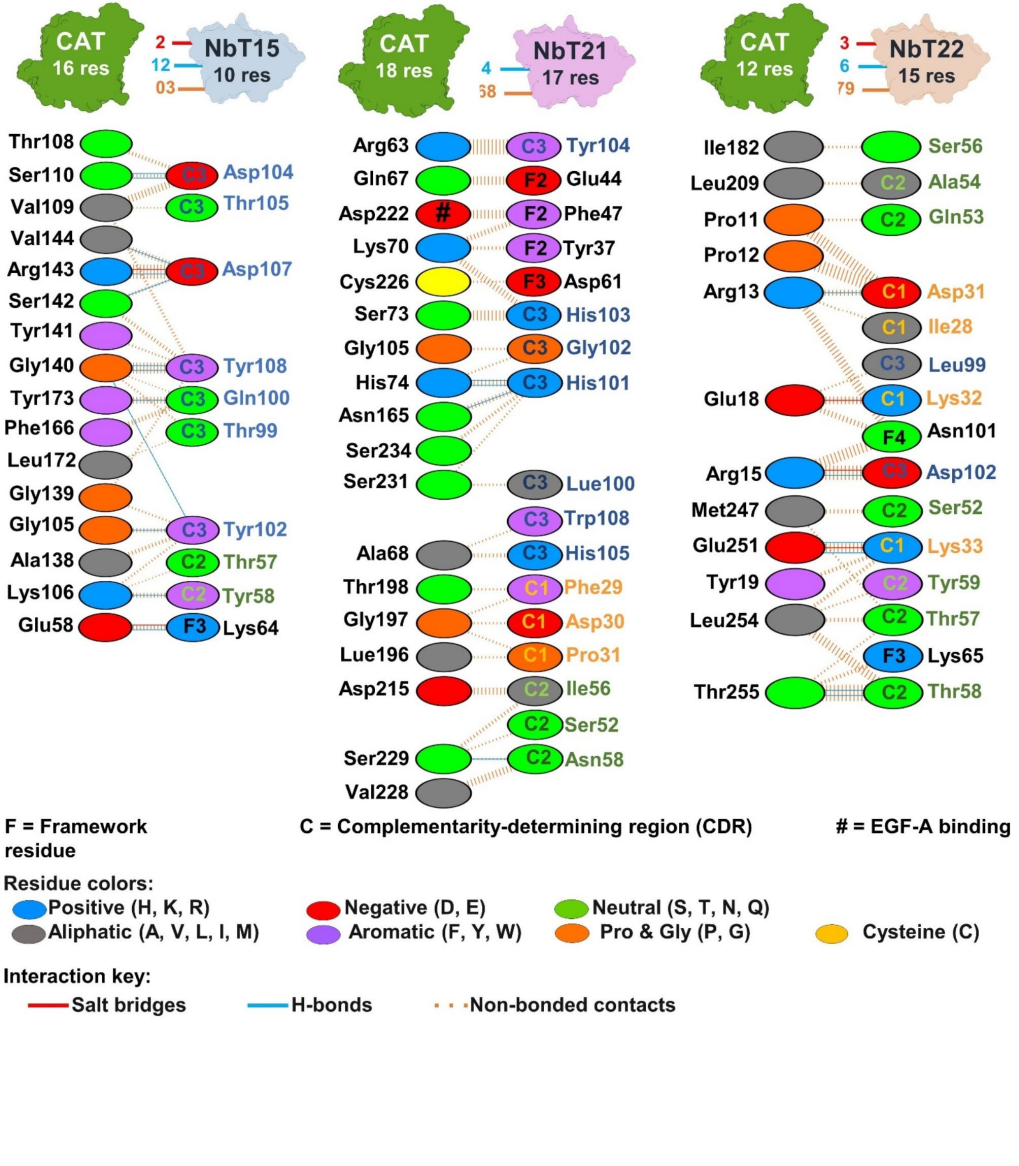
Protein–protein interactions of the wt PCSK9 with NbT15, NbT21, and NbT22.

To explore how the Nbs might inhibit the PCSK9-LDLR interaction, we performed molecular docking using the ClusPro server. From this analysis, NbT15 was predicted to form 10 interactions (2 electrostatic and 8 hydrogen bonds) with the PCSK9 CAT domain. The NbT21 clone was predicted to form a similar number of interactions (9 total; 1 electrostatic and 8 hydrogen bonds) but not with any of the same residues on the PCSK9 CAT domain. The NbT22 clone was predicted to form the most contacts (12 total, 3 electrostatic, 7 hydrogen bonds, and 2 hydrophobic) with PCSK9 CAT domain, including an interaction with residue E18 that was also contacted by the NbT15 clone (Figs. 6 and 7). Notably, the CDRs of all Nbs contain many positive, negative, and polar amino acids, which may be the cause of their low polyreactivity^18^.

## Discussion

Here, we combined MACS-based screening of a YSD library with genetic selection using FLI-TRAP to select PCSK9 CAT domain-specific Nbs from a synthetic library containing ~ 5 × 10^8^ unique Nb clones. The single round of MACS was applied as an initial screening step to focus the Nb library to the PCSK9 target after which FLI-TRAP-mediated genetic selection was used to further focus the sublibrary to the PCSK9 catalytic domain. One advantage of FLI-TRAP is that it introduces the folding quality control (QC) mechanism of the Tat system^19^, which prevents export of misfolded and/or insoluble proteins and thus effectively eliminates false positive interactions that might arise from the non-specific interaction of misfolded and aggregated proteins. Another advantage of FLI-TRAP is that the overall resistance measured in the assay correlates with the affinity between two proteins such that higher levels of antibiotics can be used to select higher affinity binders^17^. From our experiment, a total of 7 out of 30 isolated clones were identified as positive hits based on selection at a relatively high concentration of Carb (300 µg/mL), indicating that the FLI-TRAP assay is useful for weeding out less desirable clones having weaker affinity and/or exhibiting non-specific binding. Overall, by combining MACS-based YSD with FLI-TRAP selection, we have created a reliable and rapid method for selecting recombinant antibodies from combinatorial libraries with a high rate of positive protein selection, low cost, and no need of expensive instrument. Currently, 3 Nbs have been approved for clinical use^14^ and over 80 Nb-based therapeutic candidates have entered preclinical and clinical trials over the last 2 decades^3^. With their strong physicochemical properties, high target specificity, and suitability for multivalent protein engineering, are rapidly emerging as an effective next-generation antibody platform to address challenges in traditional mAbs. Potential applications include but not limited to cancer^20,21^, blood and autoimmune disorders^22^, viral and bacterial infection diseases^23,24^, and ocular disease^25–27^.

Importantly, we were able to identify PCSK9 binders with workflow only required two rounds of biopanning and was achieved with a fully synthetic library that could be used for binder selection against arbitrary targets in the future. Our Nbs were also capable of inhibiting the interaction between LDLR and both PCSK9 and PCSK9^D374Y^. The molecular docking predicted that the NbT21 CDRs are located near the epidermal growth factor-like repeat A (EGF(A)) binding region on PCSK9, which might be important for disruption of the binding between PCSK9 and EGF(A) domain of LDLR. As reported in previous studies, the CAT domain of PCSK9 interacts with the EGF(A) domain of LDLR via critical amino acids, including R194, D328, D374, T377 and F379^28^. The interaction between R194 (PCSK9) and D310 (LDLR) is important for stabilizing the LDLR Ca^2+^ binding domain, which is itself important for stabilizing the PCSK9-LDLR interaction^29^. Interestingly, the predicted NbT21 binding site on PCSK9 is located next to the LDLR binding site which might explain how Nbt21 inhibits the PCSK9-LDLR interaction. Moreover, residue H101 in NbT21 was predicted to interact with H226 in PCSK9, an important location for subtilase activity and a pro-domain binding region. The molecular docking results predict that the binding sites for NbT15 and NbT22 are located opposite to the LDLR binding site. Based on this prediction, we suspect that the long, protruding CDR3s of the Nbs might bind to the CAT domain cavity, possibly interfering with its conformation and thereby hindering the PCSK9-LDLR interaction (Fig. 7d). Other binding scenarios not predicted by our docking studies may be possible. Epitope mapping studies would be needed in the future to confirm unequivocally that this is where/how binding occurs. Owing to their ability to bind and inhibit PCSK9, these Nbs may be promising therapeutic candidates for treatment or prevention of hypercholesterolemia. It should be noted that evolocumab and alirocumab are FDA-approved mAb drugs that are currently used in the treatment of hypercholesterolemia^30,31^. However, mAbs drugs are expensive due in large part to the high manufacturing costs^29^. It is possible, therefore, that VHH-based inhibitors of PCSK9 such as those isolated here could be developed less expensively through the use of microbial expression hosts or even cell-free expression platforms^32,33^.

## Methods

### Selection of PCSK9-targeting synthetic nbs via yeast display

Purified biotinylated-human PCSK9 protein was purchased from Sino Biological Inc. (China), and the yeast Nb library was obtained from McMahon et al.^14^. PCSK9-specific Nbs were first selected using a yeast display method, which was adapted from published protocols^14^. Briefly, the yeast Nb library stock was grown in Yglc media (pH 4.5; 80 mM sodium citrate [pH 4.5], 6.7 g/L yeast nitrogen base w/o amino acids, 2% glucose, and 3.8 g/L Do mix-trp) at 30℃ and 200 rpm for 48–72 h. After incubation, the enriched yeast cells were subcultured in Trp-dropout Yglc media (pH 6.0; 6.7 g/L yeast nitrogen base w/o amino acids, 2% galactose, and 3.8 g/L Do mix-trp) at 25℃ and 220 rpm for 48–72 h to induce Nb expression on the yeast cell surface. After 72 h of induction, the induced yeast cells were harvested via centrifugation at 3,000 *g* and 4℃ for 10 min. Then, the harvested cells were subjected to magnetic-activated cell sorting (MACS) isolation following the EasySep™ cell separation protocol (STEM CELL Technologies Inc., Canada). After MACS, yeast cells containing PCSK9-targeting Nbs were immediately grown in Yglc media (pH 4.5) at 30℃ and 200 rpm for 72 h to increase their number. Then, the yeast plasmids were extracted by incubating 10^9^ yeast cell/mL culture with zymolase enzyme (Zymo Research) at 37 °C for 1 h to digest the yeast cell wall, followed by common plasmid extraction using a plasmid extraction kit (Macherey-Nagel, Germany).

### Construction of pDD18-Cm ssTorA-Nb library-FLAG and pDD322-Kan CAT-Bla plasmids for FLI-TRAP selection

The DNA sequence of the CAT domain of PCSK9 was synthesized with codon optimization for *E. coli* expression by GenScript, Inc. (USA) and cloned into the pDD322-Kan GCN4-Bla plasmid between the AvrII and NotI restriction sites, which was then transformed into *E. coli* strain NEB10β (New England Biolabs [NEB], USA) to create pDD322-Kan CAT-Bla. For Nb library construction, the Nb genes were PCR amplified using plasmids extracted from the first biopanning of yeast display as templates and cloned into the pDD18-Cm ssTorA-anti-GCN4-FLAG plasmid between the XbaI and SalI restriction sites to create pDD18-Cm ssTorA-Nb library-FLAG.

### Selection of PCSK9-specific nbs via FLI-TRAP

*E. coli* NEB10β cells were co-transformed with pDD322-Kan CAT-Bla and pDD18-Cm ssTorA-Nb library-FLAG or pDD18-Cm ssTorA-Ref8-FLAG (the negative control). Anti-CAT Nbs were isolated by spreading 10^−1^−10^−2^ serially diluted overnight cells containing the library or Ref8 normalized to an OD_600_ of 2.5 directly onto LB agar plates supplemented with 1% w/v L-arabinose and 300 µg/mL Carb and incubating them at 25 °C for 48 h. The Carb-resisting ability of cells that grew on the selective plates was confirmed by spot plating. Briefly, 5 µL of six consecutive ten-fold dilutions series were spotted on LB agar plates containing 0–200 µg /mL Carb and 1% w/v L-arabinose and incubated at 25 °C for 48 h.

### Expression and purification of isolated nbs using the pET28 system

The genes of the isolated Nbs were PCR amplified and cloned into the pET28a anti-GCN4-FLAG-His plasmid between the NcoI and SalI restriction sites to generate pET28a anti-PCSK9-FLAG-His, which was then transformed into *E. coli* BL21(DE3) cells (NEB). The protein was expressed by culturing cells in 1 L of LB, induction with 1 mM IPTG when the OD_600_reached 0.6–0.7, and further culturing at 20 °C with 200 rpm agitation overnight. Next, the induced cells were harvested via centrifugation, and the soluble and insoluble fractions were prepared as previously described^14^. The protein concentration of Nb containing *E. coli* lystaes were evaluated by NaNoDrop Lite (Thermo Fisher Scientific ) with UV absorbance at 280 nm. Then, 10 µg total protein of each lysate was loaded onto 10% SDS-PAGE (TGX FastCast Acrylamide Solutions; Bio-Rad) and western blotted according to standard protocols. The polyvinylidene difluoride membrane (Thermo Fisher Scientific) was probed with mouse anti-6×His-horseradish peroxidase (HRP) antibody (1:3,000; Abcam) to detect Nbs. Gel images were captured using a Gel Doc EZ Gel Documentation System (Bio-Rad).

For purification, cells were harvested via centrifugation at 6,000 *g* and 4 °C in His-flow buffer (20 mM Tris-HCl, 500 mM NaCl, 0.1% v/v Triton X-100, and 10 mM imidazole; pH 8.0) and sonicated on ice with a Sonifier® SFX150 (Branson) at 60 s intervals with 40% amplitude and 50% duty cycle. Next, the cell debris was removed by centrifugation at 13,000 *g* and 4 °C for 20 min. Then, the supernatant was collected and immediately incubated with 1 mL of Ni-NTA resin (Bio-Rad) for 1 h at 4 °C. Next, the Ni-NTA resin and supernatant mixture was loaded onto Econo-Column® Chromatography Columns (Bio-Rad), which were then washed with four column volumes (CVs) of washing buffer (20 mM Tris-HCl, 500 mM NaCl, and 20 mM imidazole; pH 8.0). The target protein was eluted with three CVs of the elution buffer (20 mM Tris-HCl, 500 mM NaCl, and 500 mM imidazole; pH 8.0). The buffer was exchanged with PBS using a 3 kDa MWCO Vivaspin column (Thermo Fisher Scientific). Protein purity was assessed by loading 5 µL of purified protein onto 10% SDS-PAGE and stained with InstantBlue® Coomassie Protein Stain (Abcam). The concentrations of the purified Nbs were determined by absorbance at 280 nm using a NaNoDrop Lite (Thermo Fisher Scientific).

### Direct binding and neutralization assays

For binding assays, purified wt PCSK9 and PCSK9^D374Y^ (Sino Biological Inc.) were diluted in ELISA coating buffer (0.05 M NaCO_3_, pH 9.6) to a final concentration of 4 µg/mL, and 50 µL of the mixture was coated onto 96-well plates (Corning) overnight at 4 °C. The next morning, the plate was blocked with 5% non-fat milk in TBS overnight at 4 °C. After washing the plates with PBST (8.1 mM Na_2_HPO4, 1.5 mM KH_2_PO_4_, 137 NaCl, and 2.7 mM KCl containing 0.1% Tween-20), 50 µL/well of 20 µg/mL of purified Nbs were added and incubated for 1 h at RT for the pre-screening ELISA while 0, 2.5, 5, 10, or 20 µg/mL of serially diluted purified Nbs (in TBS) were used to characterize NbT15, NbT21 and NbT22. After washing with TBST, 50 µL/well of anti-FLAG-HRP antibody (1:3,000; Abcam) in TBS was added and incubated for 1 h at RT. After final washes with TBST, 100 µL/well of the 1-step™ TMB ELISA substrate solution (Thermo Fisher Scientific) was added. Finally, 100 µL/well of 1M H_2_SO_4_ was added to quench the reaction, and the absorbance of the wells was measured at 450 nm (Infinite M200; Tecan Austria GmbH).

For neutralization assays, 50 µL/well of 20 nM LDLR (Sino Biological Inc.) was diluted in ELISA coating buffer and coated onto 96-well plates (Corning). Next, the plates were blocked and washed as described above. Then, a mixture of 50 µL of TBST containing 4 nM of wt PCSK9 or PCSK9^D374Y^ pre-incubated with purified NbT15, NbT21, or NbT22 at 1,000, 100, 10, 1, or 0 ng/mL at 37 °C for 1 h was added to each well and incubated at 37 °C for another 1 h. After washing, the plates were incubated with 50 µL/well of rabbit polyclonal anti-PCSK9 antibody (1:3,000; Sino Bilogical Inc.) for 1 h at RT. After final washing, the plates were incubated with 50 µL/well of goat anti-rabbit-HRP antibody (1:150,000; Sino Biological Inc.) for 1 h at RT. Finally, the absorbance readings were performed as described above.

### Molecular modeling and protein 3D structure analysis of NbT15, NbT21, and NbT22

All Nb sequences were uploaded to the SWISS-MODEL server (https://swissmodel.expasy.org) to create 3D Nb structures using model P56963.1.A as the 3D protein structure template for 3D structure prediction. The PyMOL modeling program (version 2.5) was used for molecular labeling. The interactions between NbT15, NbT21, and NbT22 with wt PCSK9 or PCSK9^D374Y^ were simulated using the ClusPro 2.0 server (https://cluspro.bu.edu/login.php), and the Discovery Studio 2.0 program was used to visualize the molecular interactions between the Nbs and their specific target.

## Data Availability

The datasets generated and/or analyzed during the current study are available in the European Nucleotide Archive (ENA) repository, [accession number: PRJEB76224], the final accession numbers will be made available 31 May 2026. Please contact Dr. Dujduan Waraho-Zhmayev if anyone needs to access the data.
